# Hemoglobin J in a patient with severe anemia, a case report from Nepal

**DOI:** 10.1016/j.amsu.2022.104703

**Published:** 2022-09-15

**Authors:** Anish Kumar Shrestha, Ashes Rijal, Kapil Belbase, Anisha Shrestha, Sangam Shah, Sharmila Chaudhary, Simin Kunwar, Sant Kumar Yadav, Roman Dhital, Pawan Gyawali

**Affiliations:** Tribhuvan University, Institute of Medicine, Maharajgunj, Kathmandu, 44600, Nepal

**Keywords:** Hemoglobin J, Alpha thalassemia, Anemia

## Abstract

**Introduction:**

Hemoglobin J is defined by a faster movement towards anode when compared with the normal hemoglobin A. Though a pathologically distinct entity from the normal HbA, it remains clinically silent due to little physiological difference as exemplified by a similar oxygen binding capacity between the two. Though cases of symptomatic HbJ have been reported, it is uncommon. Hence, further explanations should be sought in such cases.

**Case presentation:**

Our case report exemplifies the presence of an alpha thalassemia trait along with HbJ in a symptomatic case of anemia from rural Nepal.

**Discussion:**

CE-HPLC complemented by electrophoresis, is the method of choice for characterizing various hemoglobin variants including Hb J. Hb J presents as elevated P3 peak on HPLC while thalassemia is detected by the presence of eluted proteins at the retention time between 0 and 1 minutes. P3 peak up to 6% is considered normal, values 6%–12% indicates suboptimal specimen and values greater than 15% indicates Hb J.

**Conclusion:**

Variants of hemoglobin including HbJ variant is detected using HPLC technique. Mostly clinically silent, if HbJ is associated with anemia, search for a concomitant cause should be sought one of them being alpha thalassemia when iron deficiency has been ruled out by a serum iron profile.

## Highlights

• HbJ is rare variant of hemoglobin with 71 nrw variants till date.
• CE-HPLC stands as a standard for these hemoglobin variants differentiation where Hb J presents as a P3 peak.
• Anemia when associated with HbJ should be searched for concomitant hemoglobinopathy

## 1 Introduction

Hemoglobin (Hb) J derived its name as “Fast Moving Hemoglobin (FMH) due to its characteristic fast anodal movement compared to Hb A on gel electrophoresis. It results from a mutation leading to substitution of a negatively charged amino acid in the α, β, or ϒ globin chain [1]. Oscar A. Throup and his colleagues assigned the name Hemoglobin J, while studying this characteristic feature [2]. Population studies conducted showed only nine out of a total of sixty thousand [3] and one out of twenty-six hundred [4] had hemoglobin J, thus, depicting rarity of Hb J. To the best of our knowledge, this is the first case report of hemoglobin J from Nepal. However, 2 cases of Hb J were found in a prospective study done in Department of Pathology, Tribhuvan University Teaching Hospital from October 2013 to March 2015 [5]. Most of the cases are clinically silent, thus only a few cases come to light [1,4]. In contrast to this, we here present a case of Hb J leading to severe anemia in a middle-aged female. This case has been reported as per SCARE 2020 criteria [6].

## 2 Case presentation

**Fig. 1:**
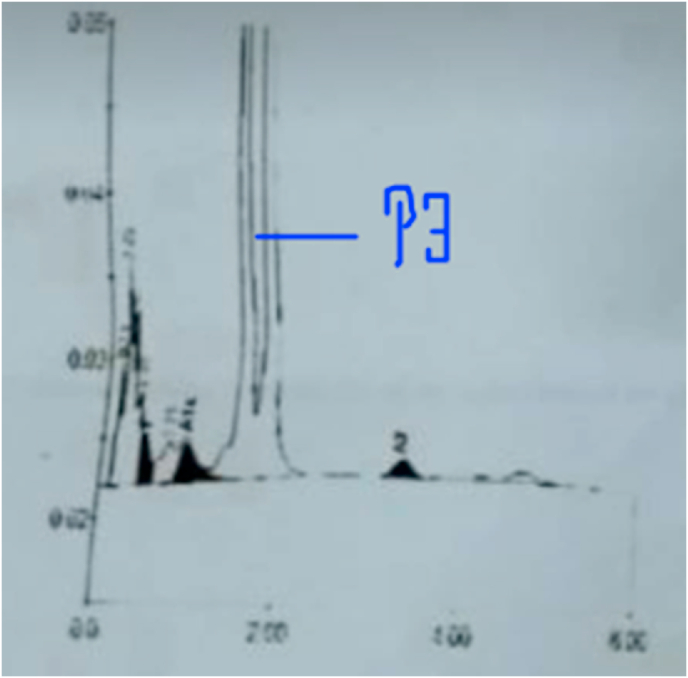
HPLC showing P3 peak.

A 23-year-old female presented to our outpatient department with complaints of palpitation, dizziness, and a burning sensation in bilateral feet for the past 20 days. She had no medical, surgical, or family history. On examination, a blood pressure of 100/60 and signs of pallor without icterus were noted. Heart and lung sounds were normal. A history of consanguineous marriage could not be elicited. Her laboratory examination revealed findings suggestive of anemia with a hemoglobin level of 6.2 gm%. On further inquiry, it was found that she was evaluated for a similar complaint about a year back. Additional blood tests revealed her MCV (mean cell hemoglobin) to be 67ft (low), an increased RDW (red cell distribution width) of 19.2% but normal total iron binding capacity (TIBC) and ferritin levels. Microscopic evaluation of her blood film showed the presence of microcytic hypochromic anemia. Her liver function tests, renal function tests, erythrocyte sedimentation rate (ESR) and Vitamin B12 levels were within the reference range. With a provisional diagnosis of anemia under evaluation, an HPLC (High Performance Liquid Chromatography) was planned which showed the finding in Fig. 1. A P3 peak was observed with a retention time of 1.51 minutes. Similarly, her hemoglobin A2 levels were also decreased (0.6%) and eluted proteins were also observed between 0 and 1 minutes. These findings were consistent with the diagnosis of HbJ-variant. Blood picture of concomitant microcytic hypochromic anemia and her HPLC report of low HbA2 levels suggest an additional diagnosis of thalassemia minor. She was managed with a blood transfusion, after which her symptoms improved. Neither HPLC of her family members nor her DNA analysis could be performed due to financial reasons of the patient.

## 3 Discussion

Hemoglobin is composed of heme made up of iron with porphyrin rings and globin chains, made up of two alpha and two non-alpha chains. Numerous variants of hemoglobin have been described which are primarily due to changes in the amino acid composition of the alpha and beta chains [7]. Hemoglobin J is one of such variants, and it itself has 71 further variants defined by changes in their amino acid composition [7]. Most cases of Hb J are clinically silent and mostly discovered incidentally during routine investigations or in conjunction with other hemoglobinopathies such as thalassemia [8] and sickle cell anemia [9] while investigating these diseases. Interestingly, there are many reports of Hb J interfering with glycated hemoglobin measurements in diabetics [[10], [11], [12]]. There are case report of Hb J being detected following transfusion as well [13]. Hence, HPLC and hemoglobin electrophoresis is suggested after a time of 12 weeks from the last transfusion. Though rare in its presentation, hemolytic anemia associated with Hb J is reported in a few instances, especially when oxidative damage precipitated an unstable form in HbJ as demonstrated by the presence of Heinz bodies in peripheral smear [14,15].

CE-HPLC complemented by electrophoresis, is the method of choice for characterizing various hemoglobin variants including Hb J(3, 4). Hb J presents as elevated P3 peak on HPLC while thalassemia is detected by the presence of eluted proteins at the retention time between 0 and 1 minutes [16]. P3 peak up to 6% is considered normal, values 6%–12% indicates suboptimal specimen and values greater than 15% indicates Hb J(3). Our patient had P3 peak of 28.9% which signifies Hb J. A Hb J of 28.9% suggests that our patient was heterozygous for mutation for Hb J as, Hb J is expressed in co-dominant form [17,18]. Literature suggests no difference in the oxygen affinity of HbJ and normal hemoglobin variant [18]. This suggests that overt symptoms should be an uncommon clinical phenomenon in this variant. Consequently, most of the literature revealed HbJ to be an asymptomatic variant. In our case, further analysis showed a blood picture of microcytic hypochromic anemia with a mild peak between 0 and 1 minutes with a low Hb A2 level in HPLC analysis, hinting towards a diagnosis of alpha thalassemia possibly an alpha thalassemia trait. However, due to limited resources, further analysis could not be performed.

## 4 Conclusion

In summary, Hb J is a rare variant of hemoglobin, the study of which is still fast, and strong till now with 71 new variants till date. Mostly clinically silent, the Hb J may at times present with hemolysis and severe anemia. CE-HPLC stands as a standard for these hemoglobin variants differentiation where Hb J presents as a P3 peak. Anemia as such, when associated with Hb J should prompt a search for a concomitant hemoglobinopathy or the presence of an unstable form. This case triggers us to think of Hb J as a potential cause when sorting out differentials for any case of anemia.

## Ethical approval

None.

## Sources of funding

None.

## Author contributions

AKS and AR wrote the original draft reviewed and edited the original manuscript. AKS, AR, KB, AS, SC, SK, SKY, RD, and PG reviewed and edited the manuscript and were in charge of the case.

## Registration of research studies

1. Name of the registry: None
2. Unique Identifying number or registration ID: None
3. Hyperlink to your specific registration (must be publicly accessible and will be checked): None

## Guarantor

Dr Kapil Belbase.

## Consent

Written informed consent was obtained from the patient for publication of this case report and accompanying images. A copy of the written consent is available for review by the Editor-in-Chief of this journal on request.

## Provenance and peer review

Not commissioned, externally peer-reviewed.

## Declaration of competing interest

None.
